# Coronavirus Disease (COVID-19) Control between Drug Repurposing and Vaccination: A Comprehensive Overview

**DOI:** 10.3390/vaccines9111317

**Published:** 2021-11-12

**Authors:** Ahmed A. Al-Karmalawy, Raya Soltane, Ayman Abo Elmaaty, Mohamed A. Tantawy, Samar A. Antar, Galal Yahya, Amani Chrouda, Rami Adel Pashameah, Muhamad Mustafa, Mobarak Abu Mraheil, Ahmed Mostafa

**Affiliations:** 1 Department of Pharmaceutical Medicinal Chemistry, Faculty of Pharmacy, Horus University-Egypt, New Damietta 34518, Egypt; 2 Department of Basic Sciences, Adham University College, Umm Al-Qura University, Makkah 21955, Saudi Arabia; rasoltan@uqu.edu.sa (R.S.); rapasha@uqu.edu.sa (R.A.P.); 3 Department of Biology, Faculty of Sciences, Tunis El Manar University, Tunis 1068, Tunisia; 4 Department of Medicinal Chemistry, Faculty of Pharmacy, Port Said University, Port Said 42526, Egypt; ayman.mohamed@pharm.psu.edu.eg; 5 Hormones Department, Medical Research and Clinical Studies Research Institute, National Research Centre, Dokki 12622, Egypt; mohamed_tantawy@daad-alumni.de; 6 Stem Cells Laboratory, Center of Excellence for Advanced Sciences, National Research Centre, Dokki 12622, Egypt; 7 Department of Pharmacology, Faculty of Pharmacy, Horus University-Egypt, New Damietta 34518, Egypt; santar@horus.edu.eg; 8 Microbiology and Immunology Department, Faculty of Pharmacy, Zagazig University, Zagazig 44519, Egypt; galalmetwally2020@gmail.com; 9 Department of Chemistry, College of Science Al-Zulfi, Majmaah University, Al-Majmaah 11932, Saudi Arabia; amain.c@mu.edu.sa; 10 Laboratory of Interfaces and Advanced Materials, Faculty of Sciences, Monastir University, Monastir 5000, Tunisia; 11 Institute of Analytical Sciences, UMR CNRS-UCBL-ENS 5280, 5 Rue la Doua, CEDEX, 69100 Villeurbanne, France; 12 Department of Medicinal Chemistry, Deraya University, Minia 61111, Egypt; muhamad_mustafa99@yahoo.com; 13 German Center for Infection Research (DZIF), Institute of Medical Microbiology, Justus-Liebig University, 35392 Giessen, Germany; mobarak.mraheil@mikrobio.med.uni-giessen.de; 14 Center of Scientific Excellence for Influenza Viruses, National Research Centre, Dokki 12622, Egypt

**Keywords:** COVID-19, SARS-CoV-2, management, vaccines, drug repurposing, clinical trials

## Abstract

Respiratory viruses represent a major public health concern, as they are highly mutated, resulting in new strains emerging with high pathogenicity. Currently, the world is suffering from the newly evolving severe acute respiratory syndrome coronavirus 2 (SARS-CoV-2). This virus is the cause of coronavirus disease 2019 (COVID-19), a mild-to-severe respiratory tract infection with frequent ability to give rise to fatal pneumonia in humans. The overwhelming outbreak of SARS-CoV-2 continues to unfold all over the world, urging scientists to put an end to this global pandemic through biological and pharmaceutical interventions. Currently, there is no specific treatment option that is capable of COVID-19 pandemic eradication, so several repurposed drugs and newly conditionally approved vaccines are in use and heavily applied to control the COVID-19 pandemic. The emergence of new variants of the virus that partially or totally escape from the immune response elicited by the approved vaccines requires continuous monitoring of the emerging variants to update the content of the developed vaccines or modify them totally to match the new variants. Herein, we discuss the potential therapeutic and prophylactic interventions including repurposed drugs and the newly developed/approved vaccines, highlighting the impact of virus evolution on the immune evasion of the virus from currently licensed vaccines for COVID-19.

## 1. Introduction

Coronaviruses comprise an enveloped capsid and positive-sense single-stranded RNA (+ssRNA) genome. They may cause respiratory and gastrointestinal disorders in a wide range of animals including birds, camels, pets, farm animals, and bats [1,2]. Coronaviruses can be classified into four main genera, namely, alpha, beta, gamma, and delta. They are all members of the order Nidovirales, family Coronaviridae, and subfamily Orthocoronaviridae [3,4,5,6].

Coronaviruses infect a variety of animal species, producing disorders in the respiratory, gastrointestinal, cardiovascular, and neurological systems. For instance, mouse hepatitis virus (MHV), rat sialodacryoadenitis virus (SDAV), avian infectious bronchitis virus (IBV), bovine coronavirus (BCV), porcine transmissible gastroenteritis virus (TGEV), turkey coronavirus (TCV), feline infectious peritonitis virus (FIPV), and rabbit coronavirus (RCV) are the prototypical coronaviruses [4]. Human coronavirus (HCoV) infection is caused by seven different viruses, which belong taxonomically to alpha “HCoV-229E” and “HCoV-NL63” and beta “HCoV-OC43”, “HCoV-HKU1”, “MERS-CoV”, and “SARS-CoV” genera [7,8].

Genetically, newly emerging SARS-CoV-2 is a descending virus from the beta-coronavirus genus. It is characterized by +ssRNA with a total length of 29.9 Kb [9]. It was proposed based on phylogenetic analysis that SARS-CoV-2 is closely linked to bat isolated SARS coronaviruses (CoVZC45 and CoVZXC2) that were collected from eastern China in 2018 (with 88% genome identity), with 79% similarity with SARS-CoV isolate and about 50% identity to MERS-CoV [10,11]. Another study depicted that SARS-CoV-2 is even more linked to another coronavirus isolate, Bat CoV RaTG13, which was isolated from the southwest part of China (92% genome identity) [11,12]. All of these outcomes highlight that bat could be the natural source of SARS-CoV-2, which is even augmented by the presence of a single open reading frame from bat-origin CoVs on gene 8 of SARS-CoV-2 [13].

The open question that has not been addressed yet is whether any other intermediate hosts between bats and humans facilitate the transmission of the virus, especially since it has been discovered that SARS-CoV-2 can infect wild animals [14]. Nevertheless, in some cases, bats can transmit viruses to humans directly, as the Nipah virus isolated in Bangladesh was transmitted via bats shedding into raw date palms sap [15].

A lot of debate has attempted to explain the origin and transmission of SARS-CoV-2. Many studies have linked the transmission of the virus from the seafood market in Wuhan, suggesting the transfer of the virus from the animal origin, “especially from bat”, to human [16]. Another study proposed transmission of the virus from another point of infection rather than the Wuhan seafood market, based on the fact that the bats are not sold in this market, and most of the genetic studies revealed that SARS-CoV-2 has a bat origin. Despite allegations that the SARS-CoV-2 virus escaped from a Chinese laboratory, analyses proved that SARS-CoV-2 is not a laboratory construct or a purposefully manipulated virus [17]. Regardless of the virus origin, all evidence confirms that SARS-CoV-2 is a zoonotic virus that has the capability and adaptive elements to easily transmit from human to human. This argument was further confirmed by the efficient silent transmission of the virus among health workers working with hospitalized patients and the emergence of the COVID-19 pandemic in a few months after the documentation of the first case in December 2019 [18,19].

Once the patient is infected, the virus needs time to establish the infection and then develop symptoms of the disease, which is called the incubation time. In the literature, there is a bit of controversy about the incubation time of SARS-CoV-2. This debate is logical, based on the fact that there are a lot of factors involved in host defense such as age, gender, and chronic diseases that modulate the onset of infection. In addition, the viral load to which the human would be exposed is another crucial factor. With the line of the aforementioned facts, the researchers have found that the incubation time of SARS-CoV-2 is ranging from 5.2 to 29 days [20,21], and the time from the onset of symptoms to death is approximately from 6 to 41 days postinfection, with an average of 14 days [20,21]. Despite many studies having revealed that the majority of COVID-19 infections are asymptomatic, most health decisionmakers rely on 14 days for quarantine, which is the longest incubation time expected based on initial observations of COVID-19 and MERS infections [22].

The scientists have assigned a unit to measure the rate of pathogen transmission from one to others causing infection, called R0, which is, by definition, the unit that measures the average number of infections resulting from one infection to completely susceptible people [23]. Based on data from previous breaks of respiratory viruses, the median R0 was indicated to be 2.4 for SARS-CoV, 1.7 for the 2009 pandemic H1N1 influenza virus, and 2.5 for SARS-CoV-2 [24]. This R0 value can be frequently changed during the pandemic, and the accurate estimation of R0 can be only assessed at the end of the pandemic. For instance, a recent study estimated a higher mean R0 value for the SARS-CoV-2 delta variant of 5.08 compared to its ancestor (average R0 = 2.79) [25,26]. Furthermore, the asymptomatic nonreported COVID-19 infections are estimated to be 40–45% of the total COVID-19 infections [19], suggesting that SARS-CoV-2 has the potential to spread silently and deeply among humans.

Regarding the transmission of SARS-CoV-2 during pregnancy, several studies demonstrated that the transplacental transmission of SARS-CoV-2 infection is possible during pregnancy. This transmission was associated with placental inflammation, neonatal viremia, and neurological symptoms [27,28]. Additionally, pregnant women are more vulnerable and likely are at greater threat of COVID-19 infection than nonpregnant women [29].

The pathogenicity and virulence determinants of SARS-CoV-2 virus is a complex and multigenic trait that is determined by several overlapping viral, environmental, and host factors [30]. Excessive exposure to anthropogenic pollutants including xenobiotics is associated with immunotoxicology risk and possible immunodeficiency in vulnerable and high-risk groups during the current COVID-19 pandemic [31]. On the other hand, the severity of COVID-19 infection is usually associated with acute and exaggerated induction of the patient’s innate immunity, leading to a cytokine storm (CS) or hypercytokinemia. This CS in critical COVID-19 patients can subsequently induce multiple-organ failure with a substantial fatality rate [32]. To this point, signaling pathways of CS were heavily studied to develop novel strategies to treat COVID-19 illness and associated inflammatory reactions. For instance, The high mobility group box 1 (HMGB1) plays a pivotal role during COVID-19 infection via mediating ACE2 expression in alveolar epithelial cells and triggering TLR4-mediated cytokine storm [33]. HMGB1 is known to be a prototypical damage-associated molecular pattern (DAMP) and a central mediator of lethal inflammation, making it a potential target for developing an antagonizing immunomodulatory agent against COVID-19-associated CS [33].

## 2. SARS-CoV-2 Life Cycle and Its Potential Targets

The upper respiratory tract (URT) is the primary site where respiratory pathogens including SARS-CoV-2 initiate infection The SARS-CoV-2 starts the respiratory COVID-19 infection via binding to the cellular angiotensin-converting enzyme 2 (ACE2) receptors [34,35]. ACE2 has a wide distribution on many tissues in the human body, as it is extensively expressed in the respiratory system (in the upper respiratory system in goblet/secretory cells in nasal epithelial cells, esophagus, and the lung in type II alveolar cells in the lower respiratory tract) [36,37]. Moreover, it is expressed in the gastrointestinal tract, especially in the ileum and colon in absorptive enterocytes, in the heart in myocardial cells, in the kidney in proximal tubule cells, and in bladder urothelial cells [37]. This explains why COVID-19 patients experienced other problems in the kidney, heart, and GIT rather than the respiratory system.

Once the binding domain (331–524 amino acid residues) of the spike glycoprotein of SARS-CoV-2 attach to the ACE2 receptor in host cells, many downstream biochemical processes are switched on to facilitate the virus entry to the host cells [38]. These downstream processes start with the fusion of the viral membrane to the host cells, leading to activation of a certain protease enzyme, which is transmembrane serine protease type II (TMPRSS2) that predominantly exists on the host cell’s surface [39]. TMPRSS2 activation leads to cutting the ACE2 and activation of receptor-attached spike proteins, which results in conformational changes and virus entry to the host cells. Therefore, the TMPRSS2 and ACE2 are the main host factors that determine the virus pathogenicity; thus, many antiviral strategies can be built based on these two factors. Once the virus enters the host cells, it releases its genetic material, which is mRNA ready for translation into main virus proteins. Moreover, the virus genome is supplemented by 14 open reading frames (ORFs), which encode both structural and nonstructural polyproteins that maintain virus integrity and virulence potentiality [40].

The nonstructural polyproteins are mainly translated from two ORFs (ORF1a and ORF1b) that produce two overlapping polyproteins (PP1a, and PP1b). Moreover, two protease enzymes, Papin-like proteases (PLpro) and chymotrypsin-like protease (3CLpro), are encoded by nsp3 and nsp5, respectively. All of these nonstructural proteins contribute significantly to virus–host interaction [39].

**Figure 1:**
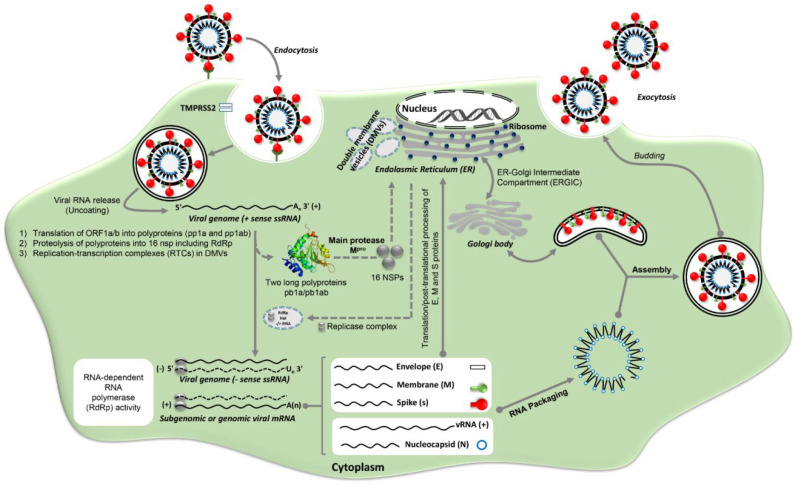
The schematic diagram illustrates the life cycle of coronavirus. The spike (S) protein of coronavirus commences infection “endocytosis” via recognizing the cellular ACE2 receptors and binding to it with its receptor-binding domain (RBD) in the S1 subunit. Following the viral genome (+ssRNA) into the host cell cytoplasm, two long polyproteins, namely pp1a and pp1ab, are translated from viral ORF1a and ORF1ab transcripts. The pp1a and pp1ab are further cleaved into 16 essential nonstructural proteins (NSPs). Necessary elements to the viral genome replication/transcription (e.g., nsp-7, nsp-8, and nsp-12) are congregated as RNA replication–transcription complexes (RTCs) inside ER-derived double-membrane vesicles (DMVs) to initiate the transcription/replication machinery for the internalized +ssRNA genome, leading to the generation of the genomic and subgenomic RNAs and their encoded viral proteins. Following the assembly of the posttranslated viral proteins and the nascent genomic RNA, the budding virion is then released from the infected host cell via exocytosis. Figure has been created by microsoft powerpoint.

Once the structural and the two polyproteins are translated, the viral genome starts to multiply; subsequently, the structural proteins including viral spike (S), membrane (M), and envelope (E) are integrated into the membrane of the endoplasmic reticulum, then into the endoplasmic reticulum–Golgi intermediate compartment (ERGIC). These proteins utilize the secretory pathway to combine with nucleocapsid (N) protein in the endoplasmic reticulum–Golgi intermediate compartment (ERGIC) membranes [41]. Eventually, the virion-containing vesicle merges with the host cell membrane to initiate the releasing process of the virus “exocytosis” (Figure 1).

Following infection of the URT and possible production of high viral load, the virus can likely disseminate to the lower respiratory tract (LRT) of the lung, causing severe complications of the infection. In terms of indications and morality, not all conditions are the same. Symptomatic instances are divided into three categories: mild, moderate, and severe, as well as asymptomatic cases [42].

**Figure 2:**
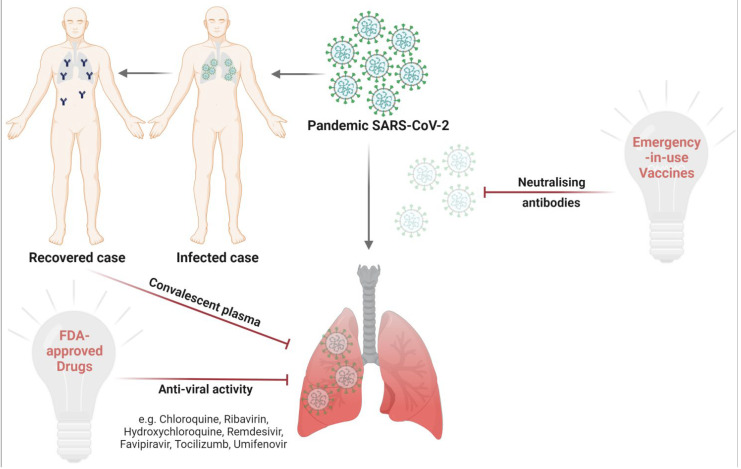
Drug repurposing for fighting COVID-19 and the introduction of different types of vaccines for prophylaxis. Figure has been created using BioRender.com.

The dramatic history of fighting COVID-19 has a lot of details including the drug repurposing [43,44,45] of different classes and the introduction of many types of emergency vaccines (Figure 2). Herein, we will discuss the aforementioned issues in a sufficiently detailed manner.

## 3. Clinical Management of COVID-19 Patients

Following infection with the SARS-CoV-2 virus, people display different responses and symptoms ranging from asymptomatic to critical [46]. Beside asymptomatic cases, symptomatic instances are divided into three categories: mild, moderate, and severe [42], demanding different clinical management.

### 3.1. Mild “Symptomatic Treatment”

COVID-19 patients with mild symptoms do not require emergency care or hospitalization, especially during this devastating pandemic that challenged the healthcare capacity of many countries. Nonetheless, all suspected or confirmed cases need to self-isolate at home and receive symptomatic treatments for fever and other mild symptoms such as cough [42]. Isolation at a hospital is recommended for individuals who are at high risk of deterioration. To this point, the decision to follow up with mild COVID-19 patients under inpatient or outpatient settings should be taken by the clinician on a case-by-case basis. At this stage, prescribing antibiotics is not recommended until there is a clinical manifestation of a secondary bacterial co-infection, which is only common in severe COVID-19 patients and is associated with fatal outcomes in COVID-19 [47].

Patients with mild COVID-19 must be closely monitored for signs of disease development. Close follow-up if medical care needs to be escalated should be provided. If patients are being treated at home, they should be instructed as well as their accompanying caregivers about complications signs and symptoms, such as trouble breathing and chest pain. The development of these symptoms demands immediate request of specific medical care services. At present, no proof makes pulse oximeter usage recommended at home [48].

### 3.2. Moderate “Pneumonia Treatment”

For COVID-19 patients with moderate illness (oxygen saturation (SpO_2_) ≥ 94% on room air at sea level), the patient should attend to the emergency room, primary care, outpatient clinic, or community service programs such home visits or telemedicine to assess his case. Patients with minor COVID-19 virus transmission should be isolated and treated according to the COVID-19 treatment protocol devised to prevent virus transmission. COVID-19 self-isolation can be done in a designated health center, a local facility, or at home [42].

Febrile patients should be examined and monitored at sites where fever may be encountered by other overlapping endemic or epidemic infections including influenza and dengue fever [49]. Patients with moderate COVID-19 infection should be treated with antipyretics for fever and discomfort, as well as adequate nourishment and water. There is no indication that the use of nonsteroidal anti-inflammatory medicines causes serious side effects in COVID-19 patients [50]. Patients with major sickness risk factors including comorbidities have to be closely monitored due to the possibility of rapid deterioration that is accompanied by lightheadedness, breathing difficulties or shallow breathing, chest pain, blue lips, and dehydration [42].

Parents of children with moderate COVID-19 should monitor the predefined health deterioration symptoms and report rapidly to the specialized physician or community outreach teams [51]. It is not recommended to use antibiotics for therapeutic or prophylactic purposes for moderate COVID-19 patients unless prescribed by a physician to treat possible secondary bacterial co-infections. Antibiotic use should be dispirited since it can lead to the evolution of antibiotic-resistance bacterial strains which can affect the sickness burden and fatalities in the populations during and after the COVID-19 pandemic era [52,53].

### 3.3. Severe and Critical Symptoms

Despite the fact that the majority of cases are developing no-to-moderate symptoms [19,54], severe COVID-19 symptoms are reported in an unprecedented number of cases that were not experienced by any health care system in the world since the 1918 “Spanish flu” [55]. According to NIH, the case is designated as severe when SpO_2_ is lower than 94% on room air at sea level and the ratio of arterial partial pressure of oxygen to fraction of inspired oxygen (PaO_2_/FiO_2_) < 300 mm Hg with a high respiratory rate (>30 breaths/min) and lung infiltrates (>50%) [46].

The development of certain complications with severe COVID-19 patients demands immediate mechanical ventilation at the hospital, and the case is referred to as “critical illness”. These complications include severe pneumonia, acute respiratory distress syndrome (ARDS) or hypoxemic respiratory failure, septic shock, cardiomyopathy and arrhythmia, acute kidney injury, thromboembolism, and nosocomial complications such as secondary bacterial infections [46]. Pulse oximeters and functional ventilation systems should be installed in every location where the critical patient can be treated. This includes outpatient clinics, emergency rooms, primary and critical care units, and informal community facilities that accommodate severe COVID-19 cases [42].

Any patient with emergency signs, as well as any patient with peripheral capillary oxygen saturation (SpO_2_) of less than 90%, should receive supplementary oxygen therapy immediately. Emergency symptoms revealed in adults such as severe respiratory distress, difficulties in breathing, shock, central cyanosis, unconsciousness, and/or convulsions, should urge them to get emergency airway care and oxygen ventilation during resuscitation to reach SpO_2_ ≥ 94% [42]. Emergency airway management, as well as oxygen therapy, should be given to children with emergency symptoms including difficulties in breathing, ARDS, cyanosis, shock, unconsciousness, or convulsions during resuscitation to achieve a SpO_2_ ≥ 94%. Once the patient is stabilized, the goal is to have a SpO_2_ level of > 90%. In young children, nasal prongs or a nasal cannula are used because they may be more tolerated [56].

In adult patients who experience excessive secretion production or retention with a weak cough, the management of airway clearance may help. Hence, gravity-assisted drainage and active breathing cycles are examples of airway clearance management tools. Mechanical insufflation–exsufflation and inspiratory positive pressure breathing are two devices that should be avoided wherever possible. Techniques should be adjusted to the particular patient and implemented according to established parameters [42]

Extrapulmonary complications such as disseminated intravascular coagulation (DIC); septic shock; acute injury of liver, heart, or kidney; or acute respiratory distress syndrome should be monitored using biochemistry laboratory testing, electrocardiogram, and chest imaging at admission and as clinically indicated [57]. The cornerstone of treatment for individuals with severe COVID-19 symptoms is the use of timely, effective, and safe supportive therapies.

COVID-19 patients should be kept an eye on regarding the signs of thromboembolic events, which can block blood flow, especially in the lungs, causing fast heart rate, chest pain, and rapid breathing [58]. A well-known coagulation parameter, D-dimer, should be monitored because the elevation of D-dimer was shown to be associated with admission to intensive care unit and high mortality rate among hospitalized COVID-19 patients [59,60].

The fetal well-being should be monitored once the pregnant woman has been resuscitated and stabilized. Based on gestational age, maternal clinical situation (hypoxia), and fetal circumstances, the frequency of fetal heart rate monitoring should be customized. Patients with COVID-19 should be treated with intravenous fluids with caution; excessive fluid resuscitation can decrease oxygenation, especially in circumstances where mechanical ventilation is limited [61].

The most catastrophic COVID-19 complication is ARDS, with higher mortality rates. Thus, fibrous stripes, ground-glass opacity, and affected lobes are common chest computed tomography (CT) results in a meta-analysis study [62]. In ARDS, hypoxemic respiratory failure is caused by an intrapulmonary ventilation–perfusion mismatch that necessitates immediate mechanical ventilation. Preoxygenation with 100% fraction of inspired oxygen (FiO_2_) for 5 minutes is recommended.

## 4. Prophylactic and Therapeutic Interventions

The number of pneumonia cases and deaths caused by COVID-19 increased dramatically all over the world. As of 27 September 2021, 232,859,879 confirmed cases of COVID-19 including 4,765,755 deaths were reported to WHO [63]. Vaccines have the upper hand to control or terminate the outbreaks and pandemics [64], especially those of viral origin [65,66]. Since the early emergence of SARS-CoV-2, following virus isolation and complete sequencing of the viral genome, efforts to develop a preventive vaccine against COVID-19 have been accelerated powered by open budgets offered by governments, multinational pharmaceutical corporations, international vaccine farms, and health organizations. From the structure point of view, SARS-CoV-2 comprises 4 fundamental structural proteins and 16 different nonstructural proteins. The structural proteins are arranged from outside to inside as follows: (1) spike glycoprotein (S), (2) small envelope glycoprotein (E), (3) membrane glycoprotein (M), and (4) nucleocapsid protein (N) [67]. In Table 1, the function of each structural and nonstructural protein and its role in virus pathogenicity have been summarized.

### 4.1. SARS-CoV-2 Vaccine Candidates

Nine months after the spark of the COVID-19 pandemic, the efforts were accelerated worldwide, enabling us to be at a point where preclinical and early clinical data were available for the currently applied vaccines. As of 22 October 2021, 128 candidate vaccines are under clinical evaluation, and 194 candidate vaccines are under preclinical evaluation, and more than 12 vaccines have completed phase 3 clinical trials and phased in WHO approval (Table 2) or received emergency licensure [73].

The main core of all approved vaccines depends on the expression of coronavirus spike protein (S) as a main immunogenic antigen of the SARS-CoV-2 virus [74], provoking the immune response to generate specific virus-neutralizing antibodies. The vaccines under evaluation in clinical trials include both traditional methods such as a purified whole-inactivated virus (17 candidate vaccines), recombinant antigen subunits containing immunogenic viral epitopes (45 candidate vaccines), live attenuated virus (2 candidate vaccines), virus-like particle vaccines (5 candidate vaccines), and next-generation vaccination platforms such as DNA- and RNA-based formulations (35 candidate vaccines), the bacterial antigen-spore specific expression vector (one candidate vaccine), and replicating or nonreplicating viral vector-based vaccines either alone (20 candidate vaccines) or with Antigen Presenting Cell (3 candidate vaccines) [64,74].

Recently, the approach of using “bacterial outer membrane vesicles (OMVs)” has emerged as a self-adjuvanted platform against respiratory viral pathogens [75,76]. A recent study has shown that the immunostimulatory pattern after immunization with recombinant RBD “rRBD” together with *N. meningitides* OMV has been significantly improved when compared with rRBD alone, declaring the development of an efficient anti-COVID-19 vaccination platform [77].

Certainly, the COVID-19 pandemic opened the gate to the clinical application of the predefined next-generation vaccination platforms, which is relatively new vaccination platforms with rare information about long-term complications and side effects such as mRNA- and adenovector vaccines [78,79,80].

The known, very rare side effects of the mRNA vaccines include myocarditis and pericarditis, whereby young men, in particular, are apparently affected after the second vaccination. Typically, the first symptoms appear within a few days after the vaccination [81].

Myocarditis is an inflammation of the heart muscle, and pericarditis is inflammation of the pericardium, the outer covering of the heart, that can result in hospitalization, heart failure, and sudden death. In both cases, the body’s immune system causes inflammation in response to an infection or other trigger. The published data show that most patients with myocarditis and/or pericarditis after vaccination with mRNA vaccines respond well to treatment and rest and feel better quickly [82,83].

Recent studies found a more than a three-fold increased risk of myocarditis in people vaccinated with mRNA-based vaccine at the age of 16 years and older compared to nonvaccinated persons (approximately 2.7 events per 100,000 persons). However, the same studies found that SARS-CoV-2 infection was associated with a significantly higher risk of myocarditis (approximately 11 events per 100,000 people) [84].

In another study, 54 vaccinated cases (≥16 years old vaccinated with BNT162b2 mRNA vaccine) met the criteria for myocarditis from more than 2.5 million vaccinated health care organization (HCO) members. Following the first dose, myocarditis was documented in 2.13 cases per 100,000. The incidence rate of myocarditis was reported to be higher (10.69 cases per 100,000) in young and adult male patients (16–29 years old) [85].

Along the same vein, several cases of unusual thrombotic events and thrombocytopenia were sporadically reported in several countries following vaccination with the recombinant adenovirus-based ChAdOx1 nCov-19 vaccine “AstraZeneca’s COVID-19 vaccine”. This thrombocytopenia is not mediated by “heparin-induced thrombocytopenia” but via generating platelet-activating antibodies against platelet-factor 4 (PF4), designated as “vaccine-induced immune thrombotic thrombocytopenia (VITT)” [86,87]. To this point, the use of ChAdOx1 nCoV-19 vaccine “AstraZeneca Vaccine”, which is an adenovector vaccine, was suspended by the European Medicines Agency (EMA) on 15 March 2021 due to frequent reporting of thrombotic events including disseminated intravascular coagulation (DIC) or arterial thrombosis. However, the vaccination with this vaccine has been resumed after the assessment of the EMA that benefits still outweigh the risks [88].

**Table 2:** Vaccines licensed for use against COVID-19 [89,90].

Approach	Vaccine Name	Vaccine Class	Manufacturer	Efficacy	Dosing and Storage
mRNA vaccine	mRNA- 1273	Encapsulated mRNA	Moderna/NIAID	94.1% against original strain	2 doses- 4 weeks apart Stored at −20 °C
	BNT162b2	Encapsulated mRNA	BioNTech/Pfizer/Fosun Pharma	95% against original strain	2 doses- 3 weeks apart Stored at −70 °C
Replication of defective viral-vector vaccine	Ad5-nCoV	Viral vector	CanSino Biological in collaboration with Beijing Institute of Biotechnology and Academy of Military Medical Sciences	Phase III (ongoing)	1 dose- Stored at 2–8 °C
	ChAdOx1 /AZD1222	Viral vector	Oxford University/AstraZeneca	70.4% against original strain	2 doses- 4 weeks apart Stored at 2–8 °C
	Sputnik-V /Gam-COVID-Vac	Viral vector	Acellena Contract Drug Research and Development in collaboration with Gamaleya Research Institute and Health Ministry of the Russian Federation	91.4% against original strain	2 doses- 3 weeks apart Stored at 2–8 °C
	JNJ-78436735/ Ad26.COV2.S	Viral vector	Johnson & Johnson	72% against original strain	1 dose- Stored at 2–8 °C
Inactivated vaccine	CoronaVac “ Sinovac”	Inactivated virus	Sinovac Research and Development Co.	50% against original strain	2 doses- 2 weeks apart Stored at 2–8 °C
	BBIBP-CorV	Inactivated virus	Beijing Institute of Biotechnology	79.34% against original strain	2 doses- 3 weeks apart Stored at 2–8 °C
	Sinopharm (Wuhan)	Inactivated virus	China National Pharmaceutical Group (Sinopharm) in collaboration with Wuhan Institute of Biological Products	undisclosed	2 doses- 3 weeks apart Stored at 2–8 °C
	BBV152/ Covaxin	Inactivated virus	Bharat Biotech	81% against original strain	2 doses- 4 weeks apart Stored at 2–8 °C
Subunit vaccine	NVX- CoV2373	Recombinant spike (rS) and Matrix-M1 proteins	Novavax	96% against original strain	2 doses- 3 weeks apart Stored at 2–8 °C
	ZF2001	The repeated dimeric form of RBD of the SARS-CoV-2 S protein	Anhui Zhifei Longcom/ Chinese Academy of Medicine	92–97%	3 doses- 4 weeks apart Stored at 2–8 °C
Virus-like particle (VLP)	CoVLP (NCT04450004)	plant-produced VLP vaccine candidate expressing SARS-CoV-2 spike protein	Medicago/GlaxoSmithKline	Phase II–III clinical trial (ongoing)	2 doses- 3 weeks apart Stored at 2–8 °C

### 4.2. Immune Response to Coronavirus Infection

The immune system has two main compartments: an innate immune response, which is fast in response with a short lifetime, and an adaptive immune response, which takes some time to be encountered with a long lifetime of protection. Concisely, as soon as SARS-CoV-2 virus infection is established, this infection is firstly encountered by the innate immune system, which is mediated by antigen-presenting cells (APCs), including dendritic cells and macrophages [91].

These APCs are characterized by the presence of pathogen recognition receptors (PRRs), which are categorized into three main types including: (1) RIG-I-like receptors (RLRs), (2) NOD-like receptors (NLRs), and (3) Toll-like receptors (TLRs). Each one of those PPRs has its mode of action based on sensing any pathogen-associated molecular patterns (PAMPs), which are any microbial product including virus glycoproteins, carbohydrate, or genetic material [91,92]. The overall outcome of the action of APCs is releasing cytokines that mediate the viral infection and stimulating the T cells and engulfing and processing the microbes for antigen presentation to the humoral immunity. For instance, many publications have depicted that activation of TLR4 by recognition of spike glycoprotein of SARS-CoV-2 will lead to activation of NF–kB and pathogen-activated protein kinases (MAPKs) via MyD88 to induce the expression of an array of proinflammatory cytokines. Recognition of RNA of SARS-CoV-2 via TLR3 will lead to activation of IRF3 and NF–kB transcription factors via TIR-domain-containing adapter-inducing interferon-*β* (TRIF) adaptor protein, which also leads to induction of many proinflammatory cytokines such as IFN-*α*, TNF-*α*, TGF-*β*, IL-1*β*, IL-6, IL-12, IL-18, IL-33, IFN-*α*, IFN-*β* [93,94]. These proinflammatory cytokines, in turn, recruit different immune modulators such as monocytes and neutrophils to the site of infection and activate other chemokines and cytokines (e.g., CCL-2, CCL-3, CCL-5, CXCL-8, CXCL-9, CXCL-10) [95]. Therefore, it is thought to be that the proinflammatory cytokines represent the first line of defense encountered by the host immune response against SARS-CoV-2 infection.

**Figure 3:**
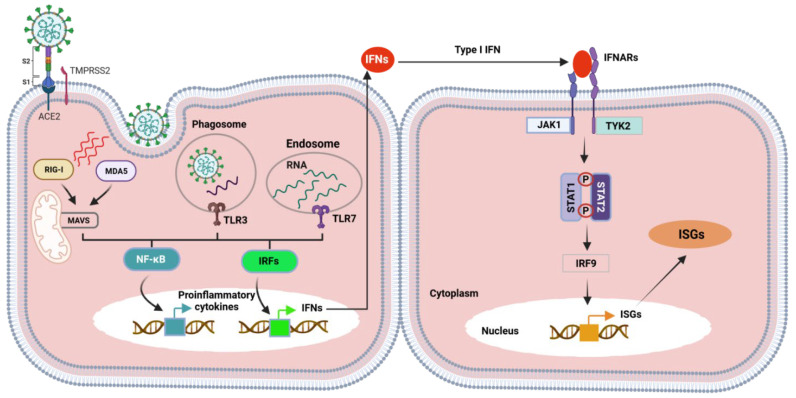
Schematic representation of the cellular innate responses against SARS-CoV-2 virus. Following the recognition of the viral PAMPs by PRRs, the downstream signaling molecules are activated. The PRR on the epithelial host cell are transmembrane localized Toll-like receptors (TLRs) and the cytosolic RIG-I-like receptors, which include the helicases: RIG-I (retinoic acid-inducible gene I), and MDA-5 (melanoma differentiation-associated gene 5). The PAMP ligand for RIG-I-like receptors is specific viral ssRNA structures with 5′-triphosphate termini (5′ppp) or long dsRNA. The TLR-3 and TLR-7 signaling pathways are subsequently activators for the IFN regulatory factors (IRFs) and the nuclear factor kappa-light-chain-enhancer of activated B cells (NF–kB), leading to proinflammatory cytokine and type-I IFN production. Following their expression, type-I IFNs induce a variety of signal transduction pathways by targeting different signaling receptors on the neighboring cell. Secreted IFN binds to a heterodimeric transmembrane receptor, composed of the subunits IFNAR1/2, to catalyze the dimerization of the IFNAR1 and IFNRA2 chains of the receptor. This dimerization stimulates the autophosphorylation of the receptor-associated tyrosine-protein kinase 1 (JAK1) and tyrosine kinase 2 (Tyk2). Subsequently, the JAK1/Tyk2 phosphorylates the downstream substrates STAT1 and STAT2. To form the essential heterotrimeric transcriptional factor complex IFN-stimulated gene factor 3 (ISGF3), active/phosphorylated STAT1 and STAT2 engage the IFN regulatory factor IRF9 in the cytoplasm. Furthermore, the ISGF3 complex localizes in the nucleus to bind the cis-element of the IFN-stimulated response element (ISRE) and stimulate the transcription of plenty of antiviral ISGs. Figure has been created using BioRender.com.

With the line of the aforementioned facts, the JAK–STAT pathway is also involved in host defense against SARS-CoV-2 infection upon activation by type 1 IFN after complexing with its receptor IFNAR (Figure 3). This activation will trigger downstream signaling ending up with the expression of IFN-stimulated genes (ISGs), which play an important role in combating viral replication and controlling the disease [96].

It is worth mentioning that excess production of proinflammatory cytokines released in response to SARC-CoV-2 infection might be a disadvantage for the host due to cell damage caused by hyperinflammation, which is called a cytokine storm, leading to Acute Respiratory Distress Syndrome (ARDS) [93,94,95].

Another important role for innate immunity is presenting the viral antigen to humoral immunity to develop memory protection against the virus infection. This cascade takes place with the aid of APC that captures and processes the virus to antigens and presents these antigens to CD4+ T helper cells via MHC I, which leads to the production of a costimulatory molecule (IL-12), which, in turn, stimulates Th1 cells initiation. Moreover, the secretion of IL-12 and INF-*α* leads to natural killer cells (NK) activation, which has a significant role in the eradication of virus-infected cells [97]. Activated Th1 cells stimulate CD8+ T cells, which can destroy SARS-CoV-2-infected cells. Later on, CD4+ T cells stimulate the cellular immune response to produce antigen-specific antibodies through activation of T-dependent B cells [46,52].

In response to infection, humoral immunity generally produces IgM and IgG antibodies. Usually, IgM can last up to 12 weeks postinfection, while IgG has a longer lifetime. Moreover, CD4+ T cells and CD8 memory are formed after virus exposure, which might protect against coronavirus for years [98,99]. Based on previous experience with coronavirus, scientists found that, after years of infection, the patient still has T cell memory cells that were able to recognize spike glycoprotein after virus exposure [100].

Following recovery of COVID-19 patients, specific IgG antibodies can last for at least 12 months in 70–90% of the convalescent COVID-19 patients who showed severe symptoms and were hospitalized [101,102,103,104,105]. The IgG titers remain in higher titers within the first 6 months of convalescence and maintain stability for the following second 6 months [101,102,103]. In mild-to-moderate patients, the IgG titers persist relatively high titers for more than 6 months after recovery [104]. Expectedly, IgA and IgM responses were less robust, and antibody titers decreased more rapidly [104,106]. On the other hand, SARS-CoV-2-specific CD4+ and CD8+ T cell responses in peripheral blood mononuclear cells (PBMCs) of convalescent COVID-19 patients for more than 10 months postinfection [107].

### 4.3. Immune-Evasion of SARS-CoV-2 and Its Emerging Variants

Generally, many respiratory systems have developed escaping mechanisms to avoid recognition by the immune system. The same is true for coronaviruses [108,109]. These immune-escape mechanisms can be developed at the different processes of the infection starting from attaching to the cells [110] until entering the host cells and establishing the infection (Table 3).

Despite that the developed and approved vaccines against COVID-19 showed promising efficacy regarding the limited numbers of the new cases or the number of cases admitted to hospitals, challenges were linked to logistic production and distribution of these potential vaccines. Furthermore, the emergence of new variants of the virus that partially or totally escape the immune response elicited by the approved vaccines requires continuous monitoring of the emerging variants to update the content of the developed vaccines or modify them totally to match the new variants.

The continuous spreading of SARS-CoV-2 among recovered and vaccinated populations prompts the virus to generate new variants of interest (VOIs) and variants of concern (VOCs), with a probable tendency to escape the preexisting virus-neutralizing antibodies [118]. Different variants have been so far documented including alpha United Kingdom variant (B.1.1.7), beta South African variant (B.1.351), gamma Brazilian variant (P.1), delta Indian variant (B.1.617.2), lambda South American variant (C.37), and mu South American variant (B.1.621) [119,120]. Meanwhile, other uncharacterized variants were detected and are currently under close monitoring as “Variants under Monitoring (VUM)” [121,122]. For instance, the emergence of the delta variant was associated with a higher transmission rate and was declared to be more contagious than its ancestor strain [25,26]. The anti-COVID-19 vaccines BNT162b2 and ChAdOx1 nCoV-19 displayed a significant reduction in effectiveness against delta variant after the first vaccine dose (30.7%) as when compared to alpha variant (48.7%). After receiving the second vaccine dose of BNT162b2, the effectiveness rates were 93.7% and 88.0% for alpha and delta variants, respectively. However, the second dose of ChAdOx1 nCoV-19 improved the effectiveness of the vaccine by 74.5% and 67.0% for alpha and delta variants, respectively [123]. This confirms that the evolution of delta variant was associated with slight differences in available vaccine effectiveness.

The SARS-CoV-2 acquires unique mutations on spike surface protein to improve the binding affinity to the cellular ACE-II receptors and to escape the neutralization of convalescent plasmas, vaccine-derived antibodies, and RDB-specific monoclonal antibodies. A recent study stated that the variants with the spike alterations D614G, A475V, and E484Q/K showed an altered sensitivity to neutralization by convalescent plasma [124]. The mutations R346S, L452R, and S477N, residing at the receptor-binding domain of the S protein, were associated with flexible interaction with the ACE2 receptor and, consequently, increased transmissibility of SARS-CoV-2 but less had affinity to neutralizing antibodies [125,126]. The alterations W152R and ΔH69-V70 were previously shown to be associated with lower sensitivity to mAbs and vaccine sera [127,128,129].

Recent studies have showed also that the spread of the delta SARS-CoV-2 variant (B.1.617.2) with higher transmission rates than the alpha variant (B.1.1.7) exhibits an impaired sensitivity to certain monoclonal and polyclonal antibodies that target non-RBD and RBD epitopes of the spike protein compared to the alpha variant. This reduced sensitivity to neutralization with the preexisting specific antibodies to spike protein has been also documented with the sera of fully vaccinated people who were immunized with Pfizer or the AstraZeneca vaccines [130]. Beta “B.1.351” and gamma “P.1” variants carrying the mutations K417N, E484K, and N501Y showed marked change in their in vitro susceptibility toward neutralizing monoclonal antibody cocktail “bamlanivimab plus Etesevimab” when compared to alpha and delta variants [131]

More recently, a new VUM lineage “C.36” and sublineages “C.36.1, C.36.2, C.36.3” have emerged and become predominant in Egypt. The transmission of this lineage and relevant sublineages was associated with reduced susceptibility to neutralization [127].

## 5. Available Therapeutic Interventions against SARS-CoV-2

Drug repurposing is a very crucial technique that provides a fast and effective solution for many uncontrollable and fast-transmitted diseases worldwide [132,133,134,135,136,137,138,139,140,141]. It provides an FDA-approved drug to be used for another new disease as an emergency plan. On the other hand, there is a big controversy in the outcome of the therapy used in treating COVID-19 patients, which might be different from one population to another and from one country to another [142]. This different treatment outcome is associated with many factors including comorbidity factors, environmental factors, and host factors [143,144]. Therefore, one drug will not fit all patients, and this fact pushes scientists to develop a more specific or group-oriented treatment against SARS-CoV-2.

### 5.1. Chloroquine and Hydroxychloroquine

It is a racemic combination of two enantiomers, R and S. Hydroxychloroquine, like chloroquine, is an aminoquinoline [145]. Hydroxychloroquine is a treatment for uncomplicated malaria, rheumatoid arthritis, chronic discoid lupus erythematosus, and systemic lupus erythematosus. In places where chloroquine susceptibility is improbable, hydroxychloroquine is frequently used for malaria prophylaxis [146]. SARS-CoV-2 prophylaxis is being researched with chloroquine and hydroxychloroquine [145]. A chloroquine derivative with a hydroxyl group, hydroxychloroquine possesses chloroquine-like pharmacokinetics, quick gastrointestinal absorption, and renal excretion, as well as a less dangerous profile [147].

Hydroxychloroquine inhibits the release of cytokines such as IL-1 and TNF-α, which is likely related to Toll-like receptor inhibition [145]. Increased pH in endosomes prevents viral particles (such as SARS-CoV and SARS-CoV-2) from fusing and entering the cell. By blocking ACE2 terminal glycosylation, hydroxychloroquine prevents SARS-CoV and SARS-CoV-2 from entering the cell. If ACE2 is not glycosylated, it may have a more difficult time interacting with the SARS-CoV-2 spike protein, further limiting viral entrance [145,148,149].

#### Clinical Trials

(a) In a randomized, placebo-controlled, and double-blind clinical trial including 821 adult participants from U.S.A. and Canada, the volunteers were in direct exposure to COVID-19 patients without any safety measures (neither face masks nor shields). On the fourth day after exposure, the participants were randomized to a placebo control group or hydroxychloroquine group. Laboratory-confirmed COVID-19 infection within 14 days was the primary outcome of the trial. Unfortunately, this clinical trial advised against the prescription of hydroxychloroquine as a postexposure prophylactic intervention for COVID-19 [150].
(b) A meta-analysis of ongoing, completed, or discontinued randomized clinical trials on the use of hydroxychloroquine or chloroquine to treat COVID-19 patients was conducted. The study ended up with a conclusion that the treatment with hydroxychloroquine is associated with increased mortality among COVID-19 patients [151].
(c) In a placebo-controlled, randomized, blind clinical trial at 34 hospitals in the U.S.A., 479 COVID-19 patients were randomized and treated either with placebo or hydroxychloroquine for 14 days. The outcomes of this randomized trial advised against the use of hydroxychloroquine in the treatment protocol of hospitalized COVID-19 adults [152].

### 5.2. Antiparasitic Drug

#### 5.2.1. Ivermectin

Ivermectin is a commonly prescribed FDA-approved antiparasitic drug. In low- and middle-income nations, it is often prescribed to treat worm infections [153]. It is indicated also to treat scabies and lice. Ivermectin is deemed extremely safe for use in humans at typical doses (0.2–0.4 mg/kg), with cumulative doses estimated equaling one-third of the present world population. Its antiviral and anti-inflammatory properties, in addition to its antiparasitic activity, have led to an ever-growing number of applications [153].

Antiviral activity of ivermectin has been demonstrated against several RNA and DNA viruses, including Zika, dengue fever, yellow fever, and others [154]. In vitro, ivermectin was found to be effective against SARS-CoV-2 via blocking the nuclear import of essential viral proteins [153]. Ivermectin showed efficient suppression of the SARS-CoV-2 replication in vitro [155]. Nevertheless, the EMA does not recommend the use of ivermectin as a routine management for the treatment of COVID-19 patients outside controlled clinical trials.

#### Clinical Trials

(a) Potential study participants were chosen at random from the state’s electronic database of patients with symptomatic, laboratory-confirmed COVID-19 during the study period. The investigation was completed by 398 (99.5%) of 400 patients including 231 women (58%) (median age 37 years) in the primary analysis population. The median time to symptoms clearance in the ivermectin group was 10 days, while the placebo group took 12 days [156]. Three weeks posttreatment, 82% of individuals using ivermectin had gotten rid of their symptoms, while 79% of those taking placebo had. The most common side effect was a headache, which was observed by 104 patients (52%) who received ivermectin and 111 patients (56%) who received a placebo [156].
(b) In another clinical trial, a total of 32 COVID-19 patients were randomized to receive the standard of care (SOC) treatment with variable doses of ivermectin (100–400 mcg/kg). Interestingly, SOC treatment plus ivermectin showed high safety and could significantly reduce symptoms and viral loads in all patients within 7 days [157].

#### 5.2.2. Nitazoxanide (NTZ)

Anti-infective thiazolidine is used to treat protozoa, helminths, anaerobic bacteria, microaerophilic bacteria, and viruses [158,159]. Thus, NTZ has a massively broad spectrum of activity, including antibacterial and anti-inflammatory characteristics, with only a few reports of side effects. Since 1996, nitazoxanide has been approved for use in most world countries. Using in silico modeling, a recent study found 73 combinations of possible 32 medications against SARS-CoV-2, which were then confirmed in vitro. The study found that nitazoxanide had a strong synergy with three antivirals: remdesivir, amodiaquine, and umifenovir. The study also found that remdesivir and hydroxychloroquine had a strong antagonistic relationship. It has been suggested that nitazoxanide be used in combination with the macrolide antibiotic azithromycin and the antimalarial medication hydroxychloroquine [160].

#### Clinical Trials

(a) Early-stage 392 symptomatic COVID-19 patients were randomized to receive NTZ (196 cases, dose: 500 mg/3 times daily/5 days) against the SOC treatment (196 cases, placebo) [161]. Interestingly, 11% improvement in symptom-free days in NTZ-treated patients was documented compared to the placebo group [161].
(b) From 20 May to 21 September 2020, a randomized clinical trial comparing NTZ (600 mg, twice) against placebo for seven days in 50 COVID-19 patients with mild respiratory insufficiency. Interestingly, a decrease in the time for hospital discharge, faster clinical swab negativity, and a significant reduction in the levels of inflammatory and lymphocyte T cells activation markers were documented among NTZ-treated patients compared to placebo [162].

#### 5.2.3. Niclosamide

Niclosamide is an anthelminthic drug that has been repurposed for use against SARS-CoV-2 after being researched [159,163]. Traditionally, niclosamide was used to cure tapeworm infections [164]. Phosphatidylethanolamine (PE) levels in infected cells were unaffected after 16 h of niclosamide treatment but significantly increased after 48 h. PE is a lipid that aids in the assembly of autophagy-inducing components, so the significant increase at this time indicates that the drug aids the autophagy machinery during viral replication [165]. By inducing autophagy and elevating PE levels while lowering the number of ether lipids and triglycerides in Vero E6 cells, niclosamide was able to reduce SARS-CoV-2 viral burden [166]. Because these lipids are required for virus production, the virus’s capacity to enter, multiply, and exit the cell is restricted.

Niclosamide was previously reported to exert antiviral activity against ssRNA viruses including coronaviruses and was suggested in 2002 for the treatment of the Severe Acute Respiratory Syndrome (SARS-CoV) outbreak. To this point, it has been proposed as a candidate antiviral drug to treat COVID-19 [164]. Due to its poor bioavailability and high cytotoxicity, the niclosamide medication is unlikely to perform effectively when applied in vitro [167].

#### Clinical Trials

(a) From 29 June to 8 August 2020, a total of 34 healthy individuals in Denmark were given a niclosamide formulation “UNI91104”, and ten were given a placebo in a placebo-controlled clinical trial. The results of this study showed that the UNI91104 is a promising and safe anti-COVID-19 drug candidate following intranasal administration [168].
(b) Another study comprised 75 COVID-19 patients who received SOC plus niclosamide in the experimental group and 75 COVID-19 patients who received only SOC therapy as a control group [169]. Within 30 days of follow-up, there was no significant difference in the incidence of death versus recovery between the two research groups. The niclosamide supplement group’s median survival time to cure was considerably shorter than the control group. After adjusting for comorbidity count, niclosamide add-on treatment increased the chance of cure by 60% each day compared to the control group [169].

### 5.3. Antibiotics

#### 5.3.1. Azithromycin

Azithromycin is a macrolide antibiotic that is frequently prescribed to treat respiratory bacterial infections and disseminated Mycobacterium avium complex (MAC) infection [170]. Common cold, influenza, and other respiratory viral diseases do not respond to antibiotics such as azithromycin, and their uncontrolled prescription for respiratory illnesses of unknown or viral etiology results in a risk of developing drug-resistant bacterial strains [171]. However, recent in vitro studies reported that azithromycin has anti-SARS-CoV-2 activity [159].

#### Clinical Trials

(a) In a clinical trial, 298 enrolled COVID-19-positive individuals between 3 June 2020 to 29 January 2021 were split into two groups: 145 were given azithromycin together with the SOC treatment, and 147 were given the SOC treatment alone [172]. This study ended up with the conclusion that, in mild-to-moderate COVID-19 patients who were managed without hospitalization, adding azithromycin to the SOC treatment did not diminish the risk of subsequent hospital admission or death.
(b) From 22 May and 30 November 2020, 2265 COVID-19 positive participants were divided into three groups: azithromycin plus SOC treatment (540 patients), SOC treatment (875 patients), and a third group that received other interventions (850 patients). The outcome of this clinical trial advised against the routine prescription of azithromycin for shortening the recovery time or reducing the risk of hospitalization for suspected COVID-19 cases [173]
(c) Through May 2020 to March 2021, 263 outpatients with SARS-CoV-2 infection were randomized into azithromycin group (n = 171) or placebo group (n = 92). Compared to the placebo group, treatment with a single dose of azithromycin (500 mg once daily for 14 days) did not lead to faster relief of the symptoms. To this point, the study did not recommend the routine use of azithromycin for COVID-19 outpatients [174].

#### 5.3.2. Fluoroquinolones

Fluoroquinolones are broad-spectrum antibacterial activity with several pharmacokinetic advantages including high oral bioavailability. Antimicrobial resistance to fluoroquinolones has increased as a result of their widespread use. Fluoroquinolones also have a high risk of major side effects (such as Clostridioides difficile infection, tendinopathy, and neuropathy) and a variety of drug–drug interactions [175]. As a result, fluoroquinolones are normally used only when the benefits clearly outweigh the risks and are licensed to treat specific bacterial infections [176].

Fluoroquinolones kill bacteria by targeting the enzymes bacterial DNA gyrase (type II topoisomerase) and topoisomerase IV, which inhibit bacterial DNA synthesis and cause bacterial DNA cleavage and death. Gram-negative and Gram-positive bacteria, anaerobes, mycobacteria, and atypical pathogens are all susceptible to fluoroquinolones. The respiratory fluoroquinolones, levofloxacin, and moxifloxacin are first-line antibiotics for the treatment of severe communicable diseases [176] and were found to exert anti-SARS-CoV-2 activity [159].

#### Clinical Trials

(a) From 15 February to 15 March 2020, 94 patients with COVID-19 including 27 severe patients at the Intensive Care Unit (ICU) and 74 ordinary patients at the general isolation ward in Wuhan Xiehe Hospital were treated with the anti-influenza drug arbidol (100 mg, three times daily for 14 days) and moxifloxacin (400 mg daily for 7–14 days) [177]. The study ended up with a conclusion that arbidol and moxifloxacin could reduce viral load and inflammation in COVID-19 patients
(b) Between 20 January and 15 March 2020, a number of 55 COVID-19 patients with mild-to-severe symptoms were hospitalized at Shenyang Sixth People’s Hospital. The treatment protocol in 53 patients included antiviral umifenovir and lopinavir/ritonavir therapies. A total of 29 patients were administered antibiotics, including moxifloxacin or linezolid. Moreover, 7 patients were treated with glucocorticoids and 9 with immunomodulators. All patients recovered, and this can partially emphasize the prophylactic administration of common antibiotics to reduce the risk of the fatal secondary bacterial co-infection [178] and their potential anti-SARS-CoV-2 activity.

### 5.4. Broad Spectrum Antivirals

#### 5.4.1. Triazavirin (TZV)

Since 2015, triazavirin has been available in Russia. TZV’s main mechanism of action is to prevent the synthetic equivalent of purine nucleoside bases from inhibiting the synthesis of viral RNA and the replication of viral genomic and subgenomic fragments [179]. The duration of key clinical influenza symptoms (intoxication, fever, and respiratory symptoms) is considerably reduced in phase II clinical trials with TZV, as well as the prevalence of opportunistic infections. However, the effectiveness of TZV against COVID-19 is uncertain [179]. The medicine was developed as a possible treatment for influenza A and B, as well as the H5N1 strain. In animal models, triazavirin was also found to be effective against two flaviviruses [180].

#### Clinical Trial

Twenty-six patients were randomly assigned to the TZV group and twenty-six patients were randomly assigned to the placebo group. The most prevalent comorbidities were hypertension (28.8%), cardiovascular disease (15.4%), diabetes (15.4%), cerebrovascular illness (7.7%), and chronic obstructive pulmonary disease (COPD) (5.8%). Seven patients in the TZV group (26.9%) and four patients in the placebo group (15.4%) stopped taking their prescriptions between the second and sixth days of the research [180].

All participants were included in the intention-to-treat analysis. The patients were 50% percent male and 58 years old on average. The time between the onset of symptoms and randomization was 7 days on average. The time to clinical improvement after TZV was 7 days, versus 12 days after placebo. Ten patients in the TZV group and six patients in the placebo group demonstrated clinical improvement in the intention-to-treat population [180].

The TZV group had a higher proportion of defervescence than the placebo group (100% versus 80%), and the median length of defervescence was 45.5 h versus 52 h in those whose body temperature was kept at 37 °C for 24 or 72 h [180].

#### 5.4.2. Umifenovir

Indole derivative with direct virucidal action and a dual mechanism inhibits numerous stages of the viral lifecycle, including virus entry, membrane effusion, and virus multiplication. In vitro hepatitis C, Ebolavirus, Zika, West Nile, and tick-borne encephalitis are all prevented [179].

#### Clinical Trial

All of the included trials yielded a total of 1052 subjects. Each study had a sample size ranging from 32 to 236 patients. All retrospective, prospective observational studies, and RCTs involving adults and umifenovir effectiveness in COVID-19 patients were included [181].

In seven studies, the time taken until the absence of viral genome in the clinical samples of COVID-19 patients was reported. There was no significant difference in negative conversion time between the umifenovir and control groups. On days 7 and 14, six studies reported a negative qRT-PCR rate, while eleven studies found a positive rate. On day 7, umifenovir was not linked to a greater negative rate. However, on day 14, umifenovir may enhance the qRT-PCR negative rate [181].

### 5.5. RNA-Dependent RNA Polymerase (RdRp) Inhibitors

#### 5.5.1. Favipiravir (FPV)

Pyrazine carboxamide derivative selectively inhibits RNA-dependent RNA polymerase from RNA viruses by imitating purines or purine nucleosides during viral replication. Favipiravir showed invariant viral activity against influenza virus, West Nile virus, Ebola virus, yellow fever virus, and Chikungunya virus, among other RNA viruses. In 2014, the treatment of a novel influenza virus in Japan was approved [179].

The mechanism of action of favipiravir is different from that of other influenza antivirals, which primarily prevent the virus from entering and exiting cells [182]. The active favipiravir-RTP stops the viral genome from reproducing by inhibiting RNA polymerase [183]. Various hypotheses exist about how favipiravir-RTP interacts with RNA-dependent RNA polymerase (RdRp) [182]. According to certain studies, favipiravir-RTP reduces RNA strand elongation and viral multiplication when it is integrated into a nascent RNA strand [182]. Purine analogs have also been shown to reduce favipiravir’s antiviral activity, implying that favipiravir-RTP and purine nucleosides compete for RdRp binding. Although favipiravir was developed to treat influenza, the RdRp catalytic domain (favipiravir’s main target) is anticipated to be similar for different RNA viruses [182].

#### Clinical Trial

At Shenzhen’s Third People’s Hospital, researchers examined the clinical efficacy of treatment for COVID-19 patients from 30 January to 4 February 2020. The purpose was to compare the clinical outcomes of individuals who received FPV treatment to those who had lopinavir/ritonavir (LPV/RTV) treatment [184]. All admitted patients to the study were assessed for eligibility. Nasopharyngeal swabs samples from people aged 16 to 75 years old tested positive for the novel coronavirus RNA; disease onset less than 7 days before enrolment; willingness to use contraception during the study and within 7 days after treatment; and no difficulty swallowing pills were among the inclusion criteria [184].

A total of 91 COVID-19 patients with laboratory confirmation who started LPV/RTV treatment between 24 January and 30 January 2020, were screened, with 45 of them being eligible for the trial’s control arm. All of the patients who were enrolled in the study completed the treatment and were observed for 14 days after it began [184]. The FPV and control groups’ baseline characteristics were compared. The baseline parameters of the two arms were not significantly different. All of the patients were in the moderate range of severity [184]. The median time for viral clearance in FPV-treated individuals was 4 days (IQR: 2.5-9), which was significantly less than the 11-day interval in the control group (IQR: 8-13).

Patients who received FPV had faster viral clearance and fewer chest CT changes than those who received LPV/RTV in this open-label comparative controlled research of COVID-19 patients [184].

#### 5.5.2. Remdesivir

RNA-dependent RNA polymerase inhibitor and adenosine nucleotide analog [185,186]. Drugs were initially developed to treat Ebola and Marburg virus infections. In vitro and in vivo action against coronaviruses such as MERS and SARS has been demonstrated in animal models [179].

#### Clinical Trial

Remdesivir was given to patients hospitalized with COVID-19, an illness caused by SARS-CoV-2 infection. The study comprised patients with proven SARS-CoV-2 infection and oxygen saturation of 94% or less when inhaling ambient air or getting oxygen supplementation [187]. Remdesivir was given to the patients in a 10-day course that included 200 mg intravenously on the first day and 100 mg daily for the next nine days. This report was based on clinical data from people who took remdesivir between 25 January 2020 and 7 March 2020 and had clinical data for at least one day later [187].

A total of 8 of the 61 individuals who received at least one dose of remdesivir had data that could not be examined (including 7 patients with no posttreatment data and 1 with a dosing error). Out of the 53 patients whose data were analyzed, 22 were from the United States, 22 from Europe or Canada, and 9 from Japan. At the start of the study, 30 patients (57%) were on mechanical breathing, and 4 (8%) were on extracorporeal membrane oxygenation.

Throughout a typical follow-up of 18 days, 36 patients (68%) improved their oxygen-support class, with 17 of 30 patients (57%) who were extubated and on mechanical ventilation improving their oxygen-support class. A total of 25 patients (47%) were released, while 7 patients (13%) died; fatality rate was 18% (6 of 34) in those who had invasive ventilation and 5% (1 of 19) in those who did not.

#### 5.5.3. Molnupiravir

Molnupiravir (EIDD-2801) is a prodrug of the active metabolite β-D-N4-hydroxycytidine (NHC, EIDD-1931). NHC shows a broad spectrum of antiviral activities against numerous positive- and negative-sense RNA viruses including influenza and coronaviruses with inhibitory concentrations 50 (IC_50_) values in the submicromolar range [188]. Despite the fact that NHC is structurally similar to remdesivir, it has increased antiviral activity against remdesivir-resistant coronavirus strains [189]. Unlike the nucleoside analog inhibitor remdesivir, NHC exerts its antiviral activity via lethal mutagenesis of the viral genome [188,190,191]. In the preclinical studies, molnupiravir could inhibit SARS-CoV-2 replication in humanized mice [192,193]. Additionally, preclinical and clinical data have shown that molnupiravir is active against the recently emerging SARS-CoV-2 variants [194].

#### Clinical Trial

In October 2021, the preliminary results of the double-blind, randomized, controlled, multicenter phase 2/3 clinical trial (NCT04575597) were declared. In this study, molnupiravir could significantly reduce the risk of hospitalization or death in nonhospitalized adults experiencing mild or moderate COVID-19. Moreover, through day 29, no death was observed in molnupiravir-treated patients, compared to eight deaths in the placebo group [195,196].

### 5.6. Protease Inhibitors

#### 5.6.1. Danoprevir

Danoprevir is a hepatitis C virus NS3 protease inhibitor that particularly inhibits HCV replication. It is combined with ritonavir to get better results. Danoprevir, in combination with ritonavir, peg-interferon alpha, and ribavirin, is now legally approved in China for the treatment of chronic hepatitis C [179].

#### Clinical Trial

From 27 January to 24 February 2020, 33 COVID-19 patients were enrolled in this trial at Nanchang’s Ninth Hospital. Patients’ clinical indices were assessed at the time of admission and discharge [197]. Different treatment strategies (danoprevir and lopinavir/ritonavir) were used to split patients into two groups. The number of days it took to attain negative qRT-PCR results, as well as the number of days spent in the hospital, were both counted and analyzed statistically. Danoprevir or lopinavir/ritonavir were administered to all COVID-19 patients who improved and were released. Blood routine, inflammation, and immune-related indices all improved considerably after treatment [197].

Patients treated with danoprevir took significantly less time to achieve negative viral shedding and had shorter hospital stays than patients treated with lopinavir/ritonavir, despite no significant differences in general information between the two groups. When taken in conjunction with danoprevir, it is an excellent therapeutic option for COVID-19 patients [197].

#### 5.6.2. Darunavir

The HIV-1 protease is inhibited by this protease inhibitor. It prevents the production of mature virus particles by blocking polypeptide cleavage in infected cells. It is used in combination with ritonavir, a potent CYP3A isozyme inhibitor, to boost a protease inhibitor’s systemic sensitivity [179].

#### Clinical Trial

This was an open-label study involving 199 hospitalized COVID-19 patients with an oxygen saturation ≤ 94% [198]. Patients were assigned in the ratio of 1:1 randomly to either receive a mixture of 400 mg lopinavir/100 mg ritonavir twice daily for two weeks or receive the standard care [198]. The efficacy of treating the lopinavir–ritonavir group was comparable to the standard care group and was not associated with significant clinical improvement or reduced mortality rates. Besides, gastrointestinal tract (GIT) adverse effects were more mutual in the lopinavir–ritonavir group. Consequently, the lopinavir–ritonavir treatment was stopped early in 13 patients due to the observed side effects [198].

### 5.7. Nucleoside Inhibitors

#### 5.7.1. Azvudine

This is a reverse transcriptase inhibitor with analogue and nucleoside azido–cytidine. The active triphosphate form is metabolized intracellularly and incorporated into the primer strand by reverse transcriptase, culminating in the termination of the g-viral DNA chain. It fights HIV, hepatitis B, and hepatitis C with antiviral characteristics [179].

#### Clinical Trial

A randomized, open-label, controlled clinical trial was carried out in China. A total of 20 COVID-19 patients participated in the study and were randomly assigned to receive either azvudine and symptomatic treatment (FNC group) or be the SOC control group [198]. For the first nucleic acid negative conversion (NANC), 10 patients in the FNC group and 10 patients in the control group had mean periods of 2.60 (SD 0.97; range 1–4) and 5.60 (SD 3.06; range 2–13) days, respectively (*p* = 0.008). The mean NANC periods for 4 newly diagnosed FNC participants and 10 newly diagnosed control individuals (starting from the initial treatment) were 2.5 (SD 1.00; range 2–4) and 9.8 (SD 4.73; range 3–19), respectively (*p* = 0.01). The FNC group had no negative effects, whereas the control group had three (*p* = 0.06). Preliminary data showed that FNC treatment can minimize NANC time when compared to standard antiviral treatment in the moderate and common COVID-19 [198].

#### 5.7.2. Tenofovir Disoproxil Fumarate

It is an adenosine nucleotide analog and DNA polymerase inhibitor that is RNA-dependent. It is licensed for the treatment of Hepatitis B and HIV-1 infection [179].

#### Clinical Trial

Between 1 February and 15 April 2020, HIV clinics at 60 Spanish hospitals treated 77 of 590 HIV-positive people who were undergoing antiretroviral therapy (ART). COVID-19 was discovered in 236 HIV-positive persons on antiretroviral therapy (ART); 151 were hospitalized, 15 were admitted to the intensive care unit, and 20 died. Men and people over the age of 70 were at higher risk of being diagnosed with COVID-19 and being admitted to the hospital. COVID-19 hospitalization was found to be 20.3% in patients on tenofovir alafenamide/emtricitabine (TAF/FTC), 10.5% in those taking tenofovir disoproxil fumarate/emtricitabine (TDF/FTC), 23.4% in those taking abacavir/lamivudine (ABC/3TC), and 20.0% in those taking other regimens [38]. There were no ICU admissions or deaths among the TDF/FTC patients. TDF/FTC-treated HIV patients had a decreased incidence of COVID-19 infection and hospitalization than those on other treatments [199].

#### 5.7.3. Ribavirin

This is a guanosine nucleoside analog and RNA polymerase virus inhibitor. It is indicated in the management of chronic hepatitis C virus [179].

#### Clinical Trial

A total of 134 patients with severe COVID-19 infection at Union Hospital, Tongji Medical College, Huazhong University of Science and Technology (Wuhan, China) were included in this study from January to February 2020 [200]. The experimental group included patients who received 500 mg of ribavirin intravenously every 12 h, whereas those who had not received ribavirin medication were in the control group. No other antiviral medications were given to the control group. Antimicrobial medication and supportive care were given to all of the patients [200].

Patients who received ribavirin and those who did not have similar baseline characteristics. After ineligible patients were excluded, the current study included 115 patients, with 71 in the control group and 44 in the therapy group. Overall, 28 (24.3%) of the 115 patients required noninvasive breathing support, while 9 (7.8%) required invasive ventilation support [200]. All of the patients were given broad-spectrum antibiotics. Ribavirin was started in the therapy group within a median of 4 days (range 1–12 days) of the SARS-CoV-2 diagnosis and 8 days (range 1–18 days) of symptom onset. Between the two groups, there were no statistically significant differences in clinical features or support measures [200].

In patients who received ribavirin, the negative conversion time for the SARS-CoV-2 test was 12.8 ± 4.1 days, compared to 14.1 ± 3.5 days in the control group. The overall mortality rate (24/115) was 20.9%. The ribavirin group had a death rate of 17.1% (7/41), and the control group had a mortality rate of 24.6% (17/69); there was no significant difference in mortality between the two groups [200]. The use of ribavirin was well-tolerated. Anemia is a common adverse effect of ribavirin therapy, and it has been observed in previous MERS-CoV and SARS-CoV infection trials.

### 5.8. Antihypertensive Drugs

Angiotensin II Receptor Blockers (ARBs) and Angiotensin-Converting Enzyme II Inhibitors (ACE Inhibitors) ACEIs

Angiotensin II receptor blockers (ARBs) have similar effects to angiotensin-converting enzyme inhibitors (ACE inhibitors), although they function through a different mechanism. Angiotensin II, a molecule that narrows blood arteries, is blocked by these medications. They assist to expand blood arteries, allowing blood to flow more freely and lowering blood pressure. People who are unable to tolerate ACE inhibitors are usually administered ARBs [201]. As illustrated in Figure 1, early evidence referred to angiotensin-converting enzyme 2 (ACE2) as a SARS-CoV-2 entry receptor. To this point, several studies have postulated the antiviral activity of ACE2 inhibitors as potential drugs to control COVID-19 [34,141,202,203].

#### Clinical Trials

(a) A total of 50,615 patients were included in 40 studies (21 cross-sectional, 2 case-control, and 17 cohorts). In subgroups by study design and considering adjusted effects, the use of ACEIs or ARBs was not linked with all-cause mortality. Disease severity was linked to the use of an ACEI or an ARB. There were no significant links discovered between the use of ACEIs or ARBs and hospital discharge, hospitalization, mechanical ventilation, length of stay, or biomarkers [204].
(b) Between 28 February and 18 August 2020, data from 228,722 veterans with a history of hypertension who had COVID-19 testing were reviewed to see if they were taking ACEIs or ARBs and the possible impact on (1) a positive COVID-19 test and (2) a severe outcome (hospitalization, mortality, and use of the intensive care unit (ICU) and/or mechanical ventilation). Interestingly, data analysis showed that ACEI use reduced the likelihood of a positive COVID-19 test in veterans with hypertension. When comparing COVID-19 inpatients to outpatients, the use of ACEI, but not ARB, was linked with a significantly higher likelihood of using mechanical ventilators [205].
(c) In this retrospective observational study, 681 COVID-19 patients who were admitted to Sina Hospital in Tehran, Iran, from 20 February to 29 May 2020, were investigated. A total of 37 were eliminated due to inadequate medical records, and 8 utilized ACEIs, leaving 636 patients in the study. In this group, 108 (17.0%) patients died, and 407 (64.0%) patients developed severe COVID-19. ARBs were given to 122 (48.0%) of the 254 individuals with hypertension (39.9%). The study found no independent connection between taking ARBs and in-hospital outcomes, except for acute kidney damage (AKI), after adjusting for known confounders, in patients with confirmed or clinically suspected COVID-19, hypertension, or nonhypertensive. The study discovered that stopping ARBs while in the hospital was linked to a higher risk of death [206].
(d) The trial enrolled 659 patients from 29 different locations around Brazil. All of the subjects were taking an ACE inhibitor or an ARB for a long time and were hospitalized with COVID-19. Patients were randomly assigned to either discontinue taking the ACEI/ARB for 30 days or continue taking it. The average number of days alive and out of the hospital for patients who stopped taking ACEI/ARBs was 21.9 days, while the average number of days alive and out of hospital for patients who remained taking these medications was 22.9 days. Between the suspending and continuing groups, the average ratio of days alive and out of the hospital was 0.95 [207]. By the end of 30 days, 91.8% of patients in the suspending ACEI/ARB group were alive and out of the hospital, compared to 95% in the continuing group [207].
(e) Another retrospective cohort study of veterans comparing the use of ARB/ACEI versus non-ARB/ACEI and ARB versus ACEI among COVID-19 outpatients and hospitalized inpatients. This observational study recommended the continuous usage of ARB or ACEI during treatment regimen for those hypertensive patients who already used them before COVID-19 infection [208].
(f) A large cohort study (8.3 million people) comprised 19486 COVID-19 patients (20–99 years old) and 1286 ICU patients. The study data showed that the ACEI and ARBs treatment was associated with reduced risks of COVID-19 disease in studied populations after adjustment of a wide range of variables. Nevertheless, the ACEI and ARBs did not reduce the risks of receiving ICU care. Interestingly, ethnic-specific effects of ACEI/ARBs on COVID-19 disease susceptibility and severity were observed [209].

### 5.9. Antiviral against Influenza Viruses

#### 5.9.1. Oseltamivir

This is a strong influenza virus neuraminidase inhibitor enzymes that hinders host cell budding, viral replication, and infection found on the virus’s surface. It is currently approved for the treatment and prevention of influenza virus A (H1N1) and influenza B infections (including pandemics) [179].

#### Clinical Trial

(a) Patients who presented with influenza-like illness (ILI) and suspected/confirmed positive for coronavirus were randomized to receive either standard therapy or standard care plus oseltamivir [210]. The most important outcome was time to recovery, which was defined as being able to resume normal activities with moderate to no fever, headache, or muscle soreness.
(b) Coronaviruses were discovered in 308 (9%) of the 3266 participants in the randomized experiment; 153 were given usual care, and 155 were given usual care plus oseltamivir. The primary result was determined in 136 and 147 people, respectively. Patients given oseltamivir had a faster recovery time: 4 days (interquartile range (IQR) 3–6) versus 5 days (IQR 3–8; hazard ratio 1.31; 95 percent confidence interval = 1.03 to1.66; *p* = 0.026) [210].

#### 5.9.2. Baloxavir Marboxil

Influenza virus polymerase activity and mRNA replication are inhibited by this selective influenza cap-dependent endonuclease inhibitor. The medication has now been approved for the treatment of influenza viruses A and B [179].

#### Clinical Trials

The antiviral activity of favipiravir and baloxavir acid against SARS-CoV-2 was investigated in vitro before the experiment began. A three-arm exploratory experiment with hospitalized adult COVID-19 patients was conducted. In a 1:1:1 ratio, patients were assigned to one of three groups: baloxavir marboxil, favipiravir, or the control group [211].

The percentage of patients who were viral-free by day 14 and the period from randomization to clinical improvement were the primary outcomes. Baloxavir acid had an EC_50_ of 5.48 µM in vitro, which was comparable to arbidol and lopinavir; however, favipiravir had no potent antiviral activity. The study enlisted the participation of thirty persons. The percentage of patients in the baloxavir marboxil, favipiravir, and control groups who were viral negative after 14 days was 70%, 77%, and 100%, respectively, with median times from randomization to clinical improvement of 14, 14, and 15 days, respectively [211].

Insufficient concentrations of these medicines compared to their antiviral activity could be one cause for their lack of virological effect and therapeutic effects. One of the study’s shortcomings is that the time between symptom onset and randomization in the baloxavir marboxil and control groups is longer than in the favipiravir group [211].

### 5.10. Immunomodulators and Neutralizing Antibodies

#### 5.10.1. Tocilizumab

Commercially available IL-6 antagonists comprise tocilizumab, siltuximab, and sarilumab. COVID-19 is being investigated for ARDS control. Tocilizumab is a recombinant humanized monoclonal antibody that targets the IL-6 receptor. Tocilizumab is used to treat giant cell arteritis (GCA), rheumatoid arthritis (RA), polyarticular juvenile idiopathic arthritis (PAJIA), systemic juvenile idiopathic arthritis (SJIA), and severe CRS caused by CAR T-cells [212]. As a bispecific T-cell engager (BiTE) product, it is also utilized to treat serious CRS. Sarilumab is an all-human monoclonal immunoglobulin G1 antibody that has been approved by the Food and Drug Administration (FDA) for the treatment of RA. It binds to both soluble and membrane-bound IL-6 receptors with high affinity. Siltuximab is a human-murine IgG monoclonal antibody that binds directly to human IL-6 and neutralizes it [212].

The treatment of Castleman disease is the only designated indication for siltuximab. Tocilizumab was the first IL-6 blocker to hit the market, and it is now widely utilized in the treatment of inflammatory illnesses. It is also worth noting that it is the only monoclonal antibody treatment for CAR T cell-induced CRS that has been approved by the FDA [212].

Tocilizumab is a recombinant monoclonal antibody against IL-6 that is now being tested in COVID-19 patients for the treatment of ARDS [213].

#### Clinical Trials

(a) Due to a lack of knowledge and clinical trials on tocilizumab, only two cases of effective COVID-19 treatment in patients with malignant comorbidities have been published [214,215].
(b) The first patient with multiple myeloma got a single dose of intravenous tocilizumab (8 mg/kg) on day 9 of hospitalization. He was given 40 mg methylprednisolone for four days before receiving tocilizumab. Despite improvements in breathing, chest tightness and CT imaging did not improve. After treatment with tocilizumab, the IL-6 blood level dropped from 122 to 21 pg/mL on day 18, and clinical symptoms and chest CT imaging both improved [216].
(c) The researchers conducted a retrospective single-center case study of 21 Chinese patients with critical (19%) and severe (81%) COVID19 infection. COVID-19 was defined as a condition requiring mechanical ventilation or organ support in the intensive care unit [217]. Tachypnea and/or respiratory failure were common in patients with severe COVID-19. The average age of the patients was 56.8 16.5 years, with 85.7% of them being men. The concentration of IL-6 was 132.4 × 278.5 pg/mL on average. All of the patients were given lopinavir, methylprednisolone, a variety of symptom relievers, and oxygen therapy. In addition to regular therapy, all patients got a single dose of intravenous tocilizumab 400 mg, and three patients received a second dose of tocilizumab 400 mg after a 12-hour break. It is worth noting that all of the patients had undergone normal treatment seven days before receiving tocilizumab; yet, there was no change in symptoms, hypoxemia, or CT imaging [217]. Tocilizumab treatment resulted in an immediate reduction in symptoms, CT opacity alterations, and hypoxemia. Within 24 h of receiving the medication, the patients’ fevers had entirely subsided. Radiological improvement in ground-glass opacities was seen in 91% of patients. The blood and oxygenation levels of 19 people were measured. The mean C-reactive protein (CRP) level reduced from 75.1 66.8 to 2.72 3.6 mg/mL on day 5 after medication [217]. Furthermore, within 5 days of treatment, patients’ oxygen saturations improved statistically considerably: 1 patient no longer required supplemental oxygen, 15 required less oxygen, 1 started the ventilator weaning process, and 2 were extubated. Finally, 19 people were discharged, with 2 remaining in the hospital in stable condition [217].
(d) Another retrospective single-center case study was conducted in 15 Chinese patients with COVID-19 who were moderately unwell (13.3%), seriously ill (40%), and critically ill (46.7%). Patients were, on average, 73 years old, with a 75% male preponderance. Diabetes mellitus, hypertension, and a prior cerebrovascular accident were among the patients’ baseline comorbidities in 27%, 60%, and 20%, respectively. All patients received tocilizumab (80600 mg) at least once, either alone (47%) or in combination with methylprednisolone (53%) [218]. Tocilizumab was administered in three doses to 33 percent of the patients. By the seventh day after treatment, 67% of patients were clinically stable, 13% had deteriorated disease, and 20% had died [218].
(e) In COVID-19, the studies also compared tocilizumab to standard care or alternative medicines that may have a favorable effect. Finally, main study endpoints such as death rate, remission of fever after 24 h, clinical improvement, biochemical response, oxygen saturation, need for mechanical ventilation, and change in Sequential Organ Failure Assessment (SOFA) score revealed significant variability [219].
(f) According to China’s National Health Commission, tocilizumab should be investigated for the treatment of COVID-19 infected people who have high IL-6 levels and substantial lung damage [220]. Tocilizumab’s adverse effects include infection, increased serum cholesterol, ALT, AST, and injection site sensitivity. Understanding the safety of tocilizumab and the potential for harm in different disorders could help clinicians determine potential COVID-19 treatment exclusion criteria. Tocilizumab has several contraindications, including known hypersensitivity to the medicine and active infection (including localized infection). Herpes zoster reactivation has been observed [221,222,223].

#### 5.10.2. Interferon β-1α

It is an interferon-like cytokine that is used to treat multiple sclerosis (MS). Mammalian cells are the ones who make it. In patients with severe COVID-19, the efficiency and safety of IFN-1 were studied. In the interferon group, 42 patients were given IFN-1α in addition to the medications prescribed by the national protocol (hydroxychloroquine plus lopinavir–ritonavir or atazanavir–ritonavir). Subcutaneous IFN-1α dosages of 44 g/mL (12 million IU/mL) were given three times weekly for two weeks.

#### Clinical Trial

A total of 92 participants were recruited between 29 February and 3 April 2020, with 42 patients in the IFN group and 39 individuals in the control group. The primary outcome, time to clinical response, was not significantly different between the IFN and control groups (9.7 5.8 versus 8.3 4.9 days, respectively, *p* = 0.95). On day 14, 66.7% of patients in the IFN group and 43.6% of patients in the control group were released, respectively. On day 28, the IFN group had a lower overall mortality rate than the control group (19% versus 43.6 percent, respectively, *p* = 0.015). The use of an early injection greatly lowered mortality (OR, 13.5; 95 percent CI, 1.5 to 118). Although including IFN in the treatment protocol did not reduce the time it took to achieve a clinical response, it did result in a greater day 14 discharge rate and a lower 28-day death rate [224].

#### 5.10.3. Baricitinib

It is a selective and reversible Janus kinase 1 (JAK1) and 2 (JAK2) inhibitor (JAK2). Janus kinases are a type of tyrosine-protein kinase that plays a key role in the proinflammatory pathway’s signaling, which is also overactive in autoimmune illnesses such as rheumatoid arthritis. Baricitinib inhibits the activation of downstream signaling molecules and proinflammatory mediators by inhibiting the activities of JAK1/2 [225].

High ferritin and IL-6 levels were linked to poor clinical outcomes in a multicenter retrospective investigation of 150 COVID-19 patients [226]. Recognizing and treating cytokine storms could be an effective way to reduce COVID-19 mortality. Because of its anti-inflammatory properties and potential off-target antiviral effects against SARS-CoV-2, baricitinib is a viable candidate [227,228].

#### Clinical Trials

Research was undertaken in Atlanta from 1 March to 18 April 2020 to investigate how baricitinib impacted the recovery of COVID-19 patients [227]. A total of 15 confirmed COVID-19 patients with moderate-to-severe symptoms were enrolled for treatment with baricitinib and hydroxychloroquine combination [227]. By the end, 12 of the 15 enrolled/treated COVID-19 patients with moderate-to-severe symptoms recovered, and 3 died. Clinical improvement following initiation of baricitinib in 11 of the 15 (73.3%) patients were temporally observed as measured by normal body temperature, reduction in inflammatory markers, elevated oxygen saturation, and recovery.

#### 5.10.4. Corticosteroid Therapy

According to a systematic review/meta-analysis evaluating the influence of corticosteroid medication on outcomes of persons with highly pathogenic coronaviruses, corticosteroids could not significantly rescue the infected cases and diminish the mortality rates, hospitalization duration, ICU admission rate, or the necessity for mechanical ventilation. They also had several negative side effects [229].

A systematic review of observational trials of corticosteroids given to SARS patients found no benefit in terms of survival or possible side effects (avascular necrosis, psychosis, diabetes, and viral clearance delay) [230,231].

In a comprehensive review of observational studies in influenza, corticosteroids were shown to increase the risk of death and future infections; the evidence was assessed as very low to low-quality due to confounding by indication [232]. After adjusting for time-varying variables, a follow-up analysis found no influence on mortality [233]. Finally, using a similar statistical technique, researchers discovered that corticosteroids had no influence on mortality but did delay MERS-CoV LRT clearance in MERS patients who were given corticosteroids [234].

Because of their lack of effectiveness and potential harm, routine corticosteroids should be avoided unless necessary. Other reasons include asthma or chronic obstructive pulmonary disease (COPD) exacerbation, septic shock, or ARDS, and each patient must undergo a risk/benefit analysis.

Corticosteroids are given to all patients with sepsis (including septic shock) [235]. According to the Surviving Sepsis guidelines [236], corticosteroids should only be given if appropriate fluids and vasopressor treatment fail to restore hemodynamic stability. Furthermore, corticosteroids may reduce mortality in individuals with moderate-to-severe ARDS, according to a recent study [237].

Clinicians considering corticosteroids for a COVID-19-positive patient with sepsis must balance the benefits of a modest reduction in mortality with the risk of prolonged coronavirus shedding in the respiratory system, as seen in MERS patients [234,238]. If corticosteroids are used, keep an eye on hyperglycemia, hypernatremia, and hypokalemia. Keep an eye out for the recurrence of inflammation and signs of adrenal insufficiency after stopping corticosteroids; these may need to be lowered. In endemic areas where steroids are utilized, diagnostic or empiric treatment should be explored because steroid medication increases the likelihood of Strongyloidiasis stercoral infection [239].

Prenatal corticosteroid treatment is indicated for women at risk of preterm birth between 24 and 34 weeks of pregnancy when there is no clinical sign of maternal infection and adequate birthing and baby care are available. The therapeutic benefits of prenatal corticosteroids may, however, exceed the risk of damage to the mother in cases where the woman has mild COVID-19. The benefits and dangers to the woman and the premature newborn should be discussed with her to ensure an informed decision, as this assessment may vary depending on the woman’s clinical condition, her and her family’s wishes, and the availability of healthcare resources [42].

#### 5.10.5. Convalescent Plasma

COVID-19 convalescent plasma, also known as “survivor’s plasma,” is blood plasma derived from patients who have recovered from COVID-19. Last year, the U.S. Food and Drug Administration issued an Emergency Use Authorization to allow the use of convalescent plasma in hospitalized patients with COVID-19.

#### Clinical Trial

Based on previous experience with convalescent plasma in Argentine hemorrhagic fever, 90 patients were included in multicenter research, of which 87 were evaluable. A total of 278 convalescent donors contributed 397 donations [240]. Patients received plasma with an IgG content of 0.7–0.8 for every 10 kg of body weight. The key goal was to make it through the first 28 days alive. In total, 77% were men, aged 54 (+/−15.6 years) (range 27–85); BMI was 29.7 +/−; 4,4; hypertension was 39%; diabetes was 20%; 19.5% had an immunosuppressive condition; and 23% worked in healthcare [240]. Plasma was infused into 55 patients (63%) who were spontaneously breathing with oxygen supplementation (mostly oxygen mask with reservoir bag in 80%) and 32 patients (37%) who were mechanically ventilated. Patients who were injected with spontaneous breathing had a 91% 28-day survival rate, compared to 63% for those who were infused with mechanical ventilation. The WHO pneumonia clinical scale improved significantly at 7 and 14 days after the infusion, as did PaO_2_/FiO_2_, ferritin, and LDH [240].

#### 5.10.6. Neutralizing Monoclonal Antibodies (mAbs) Cocktails

Specific mAbs that target specific epitopes in the RBD of SARS-CoV-2 spike protein demonstrated a clinical advantage in neutralizing the virus and controlling COVID-19 infection in household contacts of infected patients and in skilled nursing and assisted living facilities [241]. Three mAb products either in combinations “bamlanivimab/etesevimab or casirivimab/imdevimab” or mAb monotherapy “sotrovimab” have been developed and received an Emergency Use Authorization (EUA) from FDA to control mild and moderate COVID-19 infections in high-risk COVID-19 outpatients [242].

#### Clinical Trials

(a) In a randomized phase 3 clinical trial, 1035 mild-to-moderate COVID-19 patients with a high risk of severe disease development were randomized to receive either a single intravenous infusion of placebo or bamlanivimab–etesevimab mAb combination. Interestingly, the mAb cocktail resulted in an accelerated decline of SARS-CoV-2 viral loads and reduced rates of hospitalization and death.
(b) In a retrospective cohort study, 696 individuals with mild-to-moderate symptoms and treated with casirivimab–imdevimab mAb combination were compared to the untreated control group “696 mild-to-moderate COVID-19 patients” in different sites in the U.S.A. The study ended up with a conclusion that casirivimab–imdevimab mAb combination led to a significant reduction in the hospitalization rate of high-risk COVID-19 patients when compared to the control group [243].
(c) In a randomized, multicenter, phase 3 clinical trial, 583 high-risk mild-to-moderate COVID-19 patients were randomized to have 292 individuals in the control group “placebo” and 291 in sotrovimab group. At 29 days postrandomization, the study was ended and resulted in the recommendation that the sotrovimab reduced the COVID-19 progression in studied high-risk patients [244].

### 5.11. Supplementary Treatments

#### 5.11.1. Oxygen Therapy

Supplemental oxygen therapy should be started right away for patients with respiratory distress, hypoxemia, or shock, with a goal SpO_2_ of > 94% [245]. Start with 5 L/min in adults and 1–2 L/min in children using a nasal cannula. Because clinical symptoms of hypoxemia are inconsistent, SpO_2_ has to be continuously monitored. All sites where emergency oxygen is given should have pulse oximeters. In the ICU, a blood gas analyzer should be accessible to measure ventilatory parameters as well (pH, PaCo2) [56]. Using the appropriate dose (flow rate) and delivery mechanism, titrate oxygen to a goal SpO_2_ of 90% (or > 92–95% in pregnant females) [69]. In some cases of nonhypercapnic hypoxemia respiratory failure, newer high-flow oxygen systems can be used [246].

#### 5.11.2. Herbal Therapy

In epidemic and pandemic situations with nonspecific preventive and therapeutic remedies, herbal medicines with high safety indices and lower side effects are frequently followed by many patients, especially those patients with mild-to-moderate symptoms. Several herbal medicines have been assessed for their anti-SARS-CoV-2 activity either as crude extracts or as purified plant-origin components. Several herbal extracts and components could hinder SARS-CoV-2 replication in vitro and relieve COVID-19 symptoms in vivo in experimental and clinical settings such as Echinacea purpurea extract, Cuphea Ignea extract, Pomegranate Peel extract and juice (Punica Granatum), Clove (*Syzygium aromaticum* L.), and bioactive polyphenolic compounds, namely curcumin, quercetin, and hesperidin [2,45,247,248,249,250,251,252,253]. However, the main limitation of the herbal medicines is still the inconsistency of the antiviral efficacy between the cell-based in vitro assays and in vivo experimental settings due to the high hydrophilicity and low oral bioavailability of the herbal medicine components [254].

#### 5.11.3. Zinc and Vitamin Supplements

Micronutrients such as minerals, vitamins (e.g., C, D3, and B-complex), and trace elements (e.g., Zn and Fe) are crucial for maintaining a healthy immune system with its two arms: the innate and adaptive immune responses to the invading virus particles. Despite the fact that the deficiency of the predefined micronutrients could be associated with impaired immune defense in COVID-19 patients, the prescription of a high dose of zinc gluconate, vitamin C, or both did not significantly improve symptoms faster than the standard of care [255]. Nevertheless, recent studies have reported that vitamin D deficiency is strongly associated with COVID-19 clinical severity [256] and that the daily oral high dose of vitamin D3 could reduce the inflammatory markers associated with COVID-19 without any side effects [257].

## 6. Future Perspective and Conclusions

COVID-19 is a pandemic that makes researchers and scientists committed to establishing new therapeutic strategies and plans fast to conquer this devastating pandemic. One of these therapeutic strategies is developing new vaccines and using some FDA-approved drugs that could be used on a trial basis against COVID-19 and regarded as repurposed drugs. This article shows an overview that summarizes the efficacy of different groups of currently approved and candidate vaccines and repurposed drugs (e.g., antiparasitic, antimalarials, antivirals, interferons, immunomodulators, and corticosteroids) against COVID-19 infections. These repurposed drugs can be complementary to newly conditionally approved SARS-CoV-2 vaccines to attain overall mitigation of the COVID-19 pandemic. Concerning the efficacies of discussed vaccines, it has been noticed that BioNTech, Pfizer, Fosun Pharma, Moderna, NIAID vaccines may be encountered as the most efficient protective interventions to combat COVID-19. However, these currently applied vaccines are confronted with many limitations including host and viral factors. Therefore, the decision to get vaccinated with any of the approved vaccines must be taken under controlled medical supervision and in consideration of the findings of the recent clinical studies. Additionally, regarding the discussed repurposed drugs, it is worth mentioning that chloroquine, hydroxychloroquine, triazavirin, favipiravir, remdesivir, azvudine, TDF, and tocilizumab could be appraised as the most favorable repurposed drugs based on clinical trials and case reports previously debated. Finally, we hope that countries around the world, regardless of political orientations, can unite and work together within the “One Health context” to quickly and successfully control the COVID-19 pandemic.

## Figures and Tables

**Table 1:** Structural and nonstructural proteins of SARS-CoV-2 and their functions.

Structural Proteins		
Protein	Function	Refs.
S (spike glycoprotein) It is a 150 KDa transmembrane protein found exposed to the outer surface of the virus	It helps in virus attachment and entry by binding with surface ACE2 receptors on the host cell. It is proteolytically cleaved into S1, “which determines host-virus infection, and cellular tropism”, and S2 unit “which controls virus fusion and entry”.	[68]
E (small envelope glycoprotein) Smallest protein in the virus	It helps in viral particle production and maturation.	[69]
M (membrane glycoprotein) Most abundant virus protein	It determines the envelope shape of the viral particle and is crucial for viral assembly.	[69]
N (nucleocapsid protein) localized in the endoplasmic reticulum–Golgi region and highly phosphorylated	It is crucial in virus replication and cellular responses to virus infection. It can make structural changes, resulting in enhancing virus infection.	[70]
Nonstructural proteins		
Nsp 1 and 3	Promoting cellular breakdown and limiting the translation of the host’s RNA, inhibiting type-I IFN signaling, and interfering with the innate immunity of infected cells.	[71,72]
Nsp 2	Binding to prohibition protein.	
Nsp 3 and 5	Elevating the expression of cytokine and the viral polyprotein cleavage.	
Nsp 4 and 6	Contributing to DMVs formation as a transmembrane scaffold protein.	
Nsp 7/8 complex	Viral RNA polymerase subunits.	
Nsp 9	RNA binding protein phosphatase.	
Nsp 10, 16, and 14	Stimulation of ExoN and 2-O-MT activity.	
Nsp 12	The main viral RNA-dependent RNA polymerase subunit.	
Nsp 13	RNA helicase, 50 triphosphatases.	
Nsp 14	Proofreading of the viral genome.	
Nsp 15	Viral endoribonuclease and chymotrypsin-like protease.	
Nsp 16	Averting MDA5 recognition and blocking cellular innate immunity.	

**Table 3:** SARS-CoV-2 mediated immune-evasion mechanisms.

Viral Component	Function	Refs.
Double membrane vesicles	Prevents recognition of the viral dsRNA by PRRs, which is an intermediate product, leads to efficient virus-induced type I interferon.	[111]
Nsp 1	Block type-I INF via host translational machinery inactivation and interfere with the phosphorylation of STAT1. Consequently, these activities lead to increased virus spreading and pathogenicity.	[112]
Nsp 3	It encodes macrodomains and papain-like protease (PLpro) (cleavage of Nsps) proteins. Both Nsp 3/PLpro proteins contribute to the escaping of SARS-CoV-2 from induced immune response and block IFN responses.	[113]
ORF3b	The protein encoded on this gene segment can interfere with the INF signaling pathway via targetting IRF3 and/or MAVs	[65,91]
ORF6	Binds to cellular karyopherin-a2 and anchoring karyopherin-b1 on internal membranes; the protein encoded on this gene segment suppresses the JAK–STAT signaling pathway, preventing nuclear translocation of the transcription factor STAT1.	[114]
ORF8	Mediates immune evasion through downregulating the expression of surface major histocompatibility complex class Ι (MHC-Ι)	[115]
Nsp 13–15 & Nsp 6	Suppress primary interferon production and interferon signaling	[116]
Nsp 14 and Nsp 16	Help in imitating the capping machinery of the host, because the viral SARS-CoV genome composition has a 50 cap less than the host cell mRNA, thus making immune system cells recognize it and induce an immune response easily. Nsp 14 starts cap formation and thereafter follows the viral RNAs cap modification by Nsp16. Consequently, the viral RNA looks similar to host cell mRNA and evades possible PRR recognition.	[117]

## Data Availability

Not applicable.
